# Low-temperature redetermination of aqua­chloridotriphenyl­tin(IV)–1,10-phenanthroline (1/1)

**DOI:** 10.1107/S1600536808024343

**Published:** 2008-08-06

**Authors:** Seik Weng Ng

**Affiliations:** a Department of Chemistry, University of Malaya, 50603 Kuala Lumpur, Malaysia

## Abstract

The crystal structure of the title compound, [Sn(C_6_H_5_)_3_Cl(H_2_O)]·C_12_H_8_N_2_, which was refined in the triclinic space group *P* 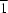 [Fu, Gao, Ma & Zhang (2005 ▶). *Chin. J. Synth. Chem.* 13, 55–57], has been redetermined in the monoclinic space group *C*2/*c* from low-temperature diffraction measurements. The Sn atom is five-coordinate in a *trans*-C_3_SnClO trigonal-bipyramidal geometry; the coordinated water mol­ecule forms a pair of hydrogen bonds to the nitro­gen heterocycle.

## Related literature

For a description of the title compound in the triclinic space group *P* 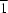, see: Fu *et al.* (2005 ▶). Aqua­chlorido­tri(*p*-chloro­phen­yl)tin^.^1,10-phenanthroline exists as a hydrogen-bonded dinuclear compound, see: Ng & Kumar Das (1996 ▶). This study also mentions the existence of a monoclinic *P*2_1_/*c* modification of the title compound. This modification is, in fact, commensurately modulated; see: Rae *et al.* (2005 ▶).
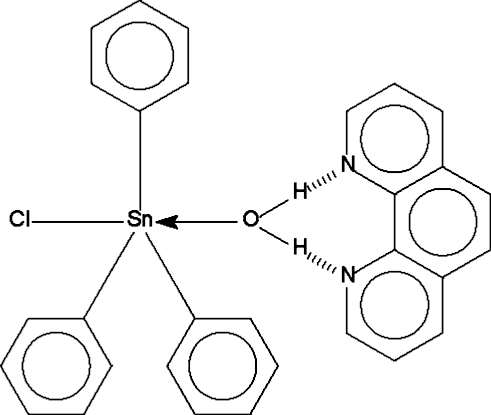


## Experimental

### 

#### Crystal data

[Sn(C_6_H_5_)_3_Cl(H_2_O)]·C_12_H_8_N_2_
*M* *_r_* = 583.66
Monoclinic, 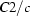

*a* = 16.3739 (2) Å
*b* = 17.3120 (2) Å
*c* = 18.4295 (2) Å
β = 105.602 (1)°
*V* = 5031.6 (1) Å^3^
*Z* = 8
Mo *K*α radiation
μ = 1.15 mm^−1^
*T* = 100 (2) K
0.30 × 0.20 × 0.10 mm

#### Data collection

Bruker SMART APEX diffractometer
Absorption correction: multi-scan (*SADABS*; Sheldrick, 1996 ▶) *T*_min_ = 0.768, *T*_max_ = 0.894
23117 measured reflections
5746 independent reflections
5333 reflections with *I* > 2σ(*I*)
*R*_int_ = 0.015

#### Refinement

*R*[*F* ^2^ > 2σ(*F* ^2^)] = 0.020
*wR*(*F* ^2^) = 0.052
*S* = 1.01
5746 reflections
324 parameters
2 restraints
H atoms treated by a mixture of independent and constrained refinement
Δρ_max_ = 0.72 e Å^−3^
Δρ_min_ = −0.60 e Å^−3^

### 

Data collection: *APEX2* (Bruker, 2007 ▶); cell refinement: *SAINT* (Bruker, 2007 ▶); data reduction: *SAINT*; program(s) used to solve structure: *SHELXS97* (Sheldrick, 2008 ▶); program(s) used to refine structure: *SHELXL97* (Sheldrick, 2008 ▶); molecular graphics: *X-SEED* (Barbour, 2001 ▶); software used to prepare material for publication: *publCIF* (Westrip, 2008 ▶).

## Supplementary Material

Crystal structure: contains datablocks global, I. DOI: 10.1107/S1600536808024343/tk2288sup1.cif

Structure factors: contains datablocks I. DOI: 10.1107/S1600536808024343/tk2288Isup2.hkl

Additional supplementary materials:  crystallographic information; 3D view; checkCIF report

## Figures and Tables

**Table 1:** Hydrogen-bond geometry (Å, °)

*D*—H⋯*A*	*D*—H	H⋯*A*	*D*⋯*A*	*D*—H⋯*A*
O1—H1*o*⋯N1	0.84 (1)	1.91 (1)	2.716 (2)	159 (3)
O1—H2*o*⋯N2	0.84 (1)	2.03 (2)	2.757 (2)	144 (3)

**Fig. 1.**
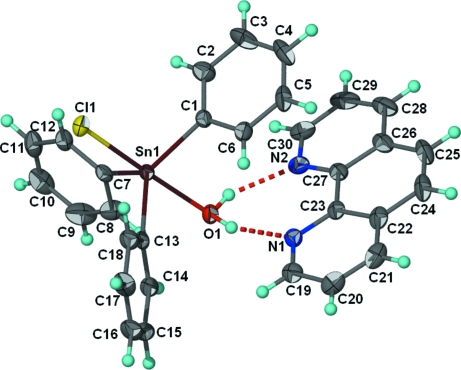
Thermal ellipsoid plot (Barbour, 2001) plot of monoclinic SnCl(H2O)(C6H5)3.C12H8N2 at the 70% probability level. Hydrogen atoms are drawn as spheres of arbitrary radius. Hydrogen bonds are shown as dashed lines.

## supplementary crystallographic information

### Comment

Interest in aquachlorotriphenyltin^.^1,10-phenanthroline (Scheme I) involves the existence of a monoclinic *P*2_1_/*c* modification [11.960 (1), 12.220 (1), 17.854 (1) Å, β 92.41 (1) °] (Ng & Kumar Das, 1996) that is, in fact, commensurately modulated in *P*2_1_/*n* [21.1053 (5), 12.2347 (3), 51.772 (2) Å,101.525 (2) °] (Rae *et al.*, 2005). The compound has the coordinated water molecules of two aquachlorotriphenyltin entities each forming hydrogen bonds to two *N*-heterocycles. A reported triclinic modification (Fu *et al.*, 2005) has an unit cell [*P*1: 12.064 (4), 12.075 (4), 18.603 (6) Å, 89.562 (6), 99.567 (5), 72.702 (5) °; *V* 2672 (5) Å^3^] that readily transforms to a monoclinic *C*2/*c* unit cell. In the correct symmetry, the tin atom is five-coordinate in a *trans*-C_3_SnClO trigonal bipyramidal geometry; the coordinated water molecule forms a pair of hydrogen bonds to one *N*-heterocycle only (Fig. 1, Table 1).

### Experimental

Triphenyltin chloride (0.39 g, 1 mmol) and 1,10-phenanthroline monohydrate (0.20 g, 1 mmol) were dissolved in hot ethanol (10 ml). Crystals of the compound separated after a day.

### Refinement

The carbon-bound hydrogen atoms were placed at calculated positions with C—H = 0.95 Å, and with *U*(H) = 1.2*U*_eq_(C). The water H-atoms were refined with a distance restraint of O—H 0.84±0.01 Å.

### Crystal data

**Table:**

[Sn(C_6_H_5_)_3_Cl(H_2_O)]·C_12_H_8_N_2_	*F*_000_ = 2352
*M**_r_* = 583.66	*D*_x_ = 1.541 Mg m^−^^3^
Monoclinic, *C*2/*c*	Mo *K*α radiation λ = 0.71073 Å
Hall symbol: -C 2yc	Cell parameters from 9047 reflections
*a* = 16.3739 (2) Å	θ = 2.3–28.3º
*b* = 17.3120 (2) Å	µ = 1.15 mm^−^^1^
*c* = 18.4295 (2) Å	*T* = 100 (2) K
β = 105.602 (1)º	Block, colorless
*V* = 5031.6 (1) Å^3^	0.30 × 0.20 × 0.10 mm
*Z* = 8	

### Data collection

**Table:**

Bruker SMART APEX diffractometer	5333 reflections with *I* > 2σ(*I*)
Radiation source: fine-focus sealed tube	*R*_int_ = 0.015
Monochromator: graphite	θ_max_ = 27.5º
ω scans	θ_min_ = 1.8º
Absorption correction: Multi-scan(SADABS; Sheldrick, 1996)	*h* = −21→21
*T*_min_ = 0.768, *T*_max_ = 0.894	*k* = −22→22
23117 measured reflections	*l* = −22→23
5746 independent reflections	

### Refinement

**Table:**

Refinement on *F*^2^	Secondary atom site location: difference Fourier map
Least-squares matrix: full	Hydrogen site location: inferred from neighbouring sites
*R*[*F*^2^ > 2σ(*F*^2^)] = 0.020	H atoms treated by a mixture of independent and constrained refinement
*wR*(*F*^2^) = 0.052	*w* = 1/[σ^2^(*F*_o_^2^) + (0.0231*P*)^2^ + 10.9613*P*] where *P* = (*F*_o_^2^ + 2*F*_c_^2^)/3
*S* = 1.01	(Δ/σ)_max_ = 0.001
5746 reflections	Δρ_max_ = 0.72 e Å^−^^3^
324 parameters	Δρ_min_ = −0.60 e Å^−^^3^
2 restraints	Extinction correction: none
Primary atom site location: structure-invariant direct methods	

### Fractional atomic coordinates and isotropic or equivalent isotropic displacement parameters (Å^2^)

**Table:**

	*x*	*y*	*z*	*U*_iso_*/*U*_eq_	
Sn1	0.283489 (7)	0.505787 (6)	0.562615 (6)	0.01482 (4)	
Cl1	0.18859 (3)	0.44885 (2)	0.44433 (2)	0.02027 (8)	
O1	0.37284 (8)	0.56254 (7)	0.67064 (7)	0.0195 (2)	
H1O	0.3578 (17)	0.6082 (8)	0.6762 (16)	0.050 (8)*	
H2O	0.4223 (9)	0.5695 (16)	0.6674 (16)	0.053 (8)*	
N1	0.36099 (9)	0.71863 (8)	0.67875 (8)	0.0188 (3)	
N2	0.50883 (9)	0.65084 (9)	0.65883 (8)	0.0216 (3)	
C1	0.34419 (10)	0.58146 (9)	0.50254 (9)	0.0172 (3)	
C2	0.40237 (12)	0.55391 (11)	0.46606 (11)	0.0264 (4)	
H2	0.4115	0.4999	0.4636	0.032*	
C3	0.44752 (13)	0.60528 (14)	0.43296 (12)	0.0343 (5)	
H3	0.4872	0.5861	0.4081	0.041*	
C4	0.43462 (13)	0.68404 (13)	0.43620 (12)	0.0330 (5)	
H4	0.4662	0.7189	0.4146	0.040*	
C5	0.37588 (13)	0.71172 (11)	0.47086 (11)	0.0287 (4)	
H5	0.3662	0.7657	0.4723	0.034*	
C6	0.33064 (12)	0.66094 (10)	0.50385 (10)	0.0214 (3)	
H6	0.2901	0.6806	0.5275	0.026*	
C7	0.33380 (10)	0.39676 (9)	0.60801 (10)	0.0179 (3)	
C8	0.36605 (13)	0.38717 (11)	0.68536 (11)	0.0274 (4)	
H8	0.3716	0.4306	0.7178	0.033*	
C9	0.39024 (15)	0.31425 (13)	0.71554 (13)	0.0366 (5)	
H9	0.4120	0.3082	0.7685	0.044*	
C10	0.38270 (13)	0.25093 (11)	0.66890 (13)	0.0331 (5)	
H10	0.3982	0.2012	0.6898	0.040*	
C11	0.35274 (12)	0.25958 (10)	0.59211 (13)	0.0292 (4)	
H11	0.3493	0.2161	0.5600	0.035*	
C12	0.32755 (11)	0.33198 (10)	0.56158 (11)	0.0218 (3)	
H12	0.3059	0.3375	0.5086	0.026*	
C13	0.18329 (10)	0.54725 (9)	0.60574 (10)	0.0178 (3)	
C14	0.19228 (12)	0.54833 (10)	0.68337 (10)	0.0217 (3)	
H14	0.2444	0.5327	0.7171	0.026*	
C15	0.12560 (13)	0.57213 (10)	0.71168 (11)	0.0264 (4)	
H15	0.1321	0.5724	0.7645	0.032*	
C16	0.04974 (13)	0.59533 (11)	0.66251 (12)	0.0292 (4)	
H16	0.0043	0.6117	0.6818	0.035*	
C17	0.03984 (12)	0.59478 (11)	0.58568 (12)	0.0281 (4)	
H17	−0.0122	0.6110	0.5522	0.034*	
C18	0.10621 (11)	0.57037 (10)	0.55742 (11)	0.0222 (3)	
H18	0.0989	0.5694	0.5045	0.027*	
C19	0.29276 (11)	0.75240 (11)	0.68954 (10)	0.0226 (4)	
H19	0.2498	0.7204	0.6994	0.027*	
C20	0.27997 (12)	0.83268 (11)	0.68733 (11)	0.0273 (4)	
H20	0.2301	0.8543	0.6959	0.033*	
C21	0.34104 (13)	0.87859 (11)	0.67250 (11)	0.0282 (4)	
H21	0.3340	0.9331	0.6704	0.034*	
C22	0.41470 (12)	0.84523 (10)	0.66025 (10)	0.0234 (4)	
C23	0.42300 (11)	0.76392 (10)	0.66440 (9)	0.0184 (3)	
C24	0.47983 (13)	0.89168 (11)	0.64384 (11)	0.0300 (4)	
H24	0.4737	0.9463	0.6413	0.036*	
C25	0.54943 (13)	0.85914 (12)	0.63203 (11)	0.0309 (4)	
H25	0.5915	0.8910	0.6206	0.037*	
C26	0.56146 (12)	0.77707 (12)	0.63629 (10)	0.0257 (4)	
C27	0.49893 (11)	0.72867 (10)	0.65276 (9)	0.0201 (3)	
C28	0.63516 (12)	0.74201 (14)	0.62609 (11)	0.0326 (5)	
H28	0.6781	0.7727	0.6145	0.039*	
C29	0.64472 (13)	0.66405 (14)	0.63288 (11)	0.0346 (5)	
H29	0.6942	0.6395	0.6265	0.042*	
C30	0.57960 (12)	0.62074 (12)	0.64960 (11)	0.0283 (4)	
H30	0.5868	0.5664	0.6546	0.034*	

### Atomic displacement parameters (Å^2^)

**Table:**

	*U*^11^	*U*^22^	*U*^33^	*U*^12^	*U*^13^	*U*^23^
Sn1	0.01639 (6)	0.01177 (6)	0.01746 (6)	−0.00055 (4)	0.00652 (4)	−0.00002 (4)
Cl1	0.02075 (19)	0.01617 (18)	0.0221 (2)	−0.00109 (14)	0.00273 (15)	−0.00226 (14)
O1	0.0198 (6)	0.0156 (6)	0.0235 (6)	−0.0023 (5)	0.0066 (5)	−0.0015 (5)
N1	0.0187 (7)	0.0197 (7)	0.0170 (7)	−0.0021 (5)	0.0033 (6)	−0.0004 (5)
N2	0.0213 (7)	0.0260 (7)	0.0176 (7)	0.0007 (6)	0.0051 (6)	−0.0019 (6)
C1	0.0168 (8)	0.0183 (8)	0.0160 (8)	−0.0034 (6)	0.0033 (6)	0.0001 (6)
C2	0.0241 (9)	0.0281 (9)	0.0296 (10)	0.0026 (7)	0.0118 (8)	0.0030 (7)
C3	0.0222 (9)	0.0508 (13)	0.0341 (11)	−0.0009 (9)	0.0152 (8)	0.0082 (9)
C4	0.0270 (10)	0.0426 (11)	0.0272 (10)	−0.0153 (9)	0.0035 (8)	0.0114 (9)
C5	0.0363 (11)	0.0216 (9)	0.0234 (9)	−0.0116 (8)	−0.0002 (8)	0.0035 (7)
C6	0.0264 (9)	0.0188 (8)	0.0183 (8)	−0.0031 (7)	0.0046 (7)	−0.0003 (6)
C7	0.0151 (7)	0.0169 (7)	0.0240 (9)	0.0004 (6)	0.0091 (6)	0.0027 (6)
C8	0.0338 (10)	0.0258 (9)	0.0240 (9)	0.0089 (8)	0.0102 (8)	0.0037 (7)
C9	0.0460 (13)	0.0365 (11)	0.0307 (11)	0.0169 (10)	0.0161 (10)	0.0148 (9)
C10	0.0320 (10)	0.0213 (9)	0.0506 (13)	0.0112 (8)	0.0190 (10)	0.0157 (9)
C11	0.0227 (9)	0.0150 (8)	0.0517 (13)	0.0007 (7)	0.0132 (9)	−0.0005 (8)
C12	0.0180 (8)	0.0179 (8)	0.0300 (9)	−0.0011 (6)	0.0074 (7)	−0.0003 (7)
C13	0.0191 (8)	0.0112 (7)	0.0254 (9)	−0.0023 (6)	0.0102 (7)	−0.0011 (6)
C14	0.0243 (9)	0.0168 (8)	0.0260 (9)	−0.0002 (7)	0.0102 (7)	−0.0006 (6)
C15	0.0363 (10)	0.0198 (8)	0.0288 (10)	−0.0002 (7)	0.0189 (8)	−0.0015 (7)
C16	0.0291 (10)	0.0226 (9)	0.0439 (12)	0.0031 (7)	0.0235 (9)	0.0005 (8)
C17	0.0209 (9)	0.0248 (9)	0.0403 (11)	0.0038 (7)	0.0114 (8)	0.0035 (8)
C18	0.0232 (9)	0.0188 (8)	0.0264 (9)	0.0005 (6)	0.0095 (7)	0.0018 (7)
C19	0.0197 (8)	0.0270 (9)	0.0196 (9)	0.0000 (7)	0.0028 (7)	−0.0005 (7)
C20	0.0246 (9)	0.0276 (9)	0.0267 (10)	0.0069 (7)	0.0019 (8)	−0.0024 (7)
C21	0.0341 (10)	0.0200 (8)	0.0242 (9)	0.0044 (7)	−0.0030 (8)	−0.0001 (7)
C22	0.0275 (9)	0.0213 (8)	0.0168 (8)	−0.0039 (7)	−0.0018 (7)	0.0010 (6)
C23	0.0205 (8)	0.0196 (8)	0.0126 (7)	−0.0029 (6)	0.0002 (6)	0.0000 (6)
C24	0.0372 (11)	0.0223 (9)	0.0245 (10)	−0.0112 (8)	−0.0018 (8)	0.0049 (7)
C25	0.0312 (10)	0.0364 (11)	0.0219 (9)	−0.0183 (8)	0.0015 (8)	0.0057 (8)
C26	0.0227 (9)	0.0377 (10)	0.0154 (8)	−0.0088 (8)	0.0032 (7)	0.0010 (7)
C27	0.0200 (8)	0.0261 (8)	0.0134 (8)	−0.0048 (7)	0.0027 (6)	−0.0005 (6)
C28	0.0231 (9)	0.0547 (13)	0.0214 (9)	−0.0102 (9)	0.0082 (8)	−0.0015 (9)
C29	0.0236 (9)	0.0586 (14)	0.0239 (10)	0.0023 (9)	0.0104 (8)	−0.0066 (9)
C30	0.0279 (10)	0.0360 (10)	0.0211 (9)	0.0032 (8)	0.0068 (8)	−0.0055 (8)

### Geometric parameters (Å, °)

**Table:**

Sn1—C1	2.127 (2)	C12—H12	0.9500
Sn1—C7	2.138 (2)	C13—C18	1.393 (2)
Sn1—C13	2.131 (2)	C13—C14	1.398 (2)
Sn1—O1	2.346 (1)	C14—C15	1.393 (2)
Sn1—Cl1	2.5132 (4)	C14—H14	0.9500
O1—H1O	0.84 (1)	C15—C16	1.386 (3)
O1—H2O	0.84 (1)	C15—H15	0.9500
N1—C19	1.322 (2)	C16—C17	1.381 (3)
N1—C23	1.364 (2)	C16—H16	0.9500
N2—C30	1.323 (2)	C17—C18	1.391 (2)
N2—C27	1.358 (2)	C17—H17	0.9500
C1—C6	1.395 (2)	C18—H18	0.9500
C1—C2	1.390 (2)	C19—C20	1.405 (3)
C2—C3	1.397 (3)	C19—H19	0.9500
C2—H2	0.9500	C20—C21	1.361 (3)
C3—C4	1.384 (3)	C20—H20	0.9500
C3—H3	0.9500	C21—C22	1.409 (3)
C4—C5	1.376 (3)	C21—H21	0.9500
C4—H4	0.9500	C22—C23	1.414 (2)
C5—C6	1.390 (2)	C22—C24	1.432 (3)
C5—H5	0.9500	C23—C27	1.452 (2)
C6—H6	0.9500	C24—C25	1.340 (3)
C7—C12	1.398 (2)	C24—H24	0.9500
C7—C8	1.391 (3)	C25—C26	1.434 (3)
C8—C9	1.393 (3)	C25—H25	0.9500
C8—H8	0.9500	C26—C28	1.408 (3)
C9—C10	1.378 (3)	C26—C27	1.418 (2)
C9—H9	0.9500	C28—C29	1.361 (3)
C10—C11	1.376 (3)	C28—H28	0.9500
C10—H10	0.9500	C29—C30	1.404 (3)
C11—C12	1.390 (2)	C29—H29	0.9500
C11—H11	0.9500	C30—H30	0.9500
			
C1—Sn1—C7	124.25 (6)	C14—C13—Sn1	120.42 (13)
C1—Sn1—C13	120.08 (6)	C13—C14—C15	120.64 (18)
C1—Sn1—O1	84.93 (5)	C13—C14—H14	119.7
C1—Sn1—Cl1	93.24 (5)	C15—C14—H14	119.7
C7—Sn1—C13	113.94 (6)	C16—C15—C14	119.73 (18)
C7—Sn1—Cl1	94.38 (5)	C16—C15—H15	120.1
C7—Sn1—O1	87.19 (6)	C14—C15—H15	120.1
C13—Sn1—O1	84.80 (6)	C15—C16—C17	120.38 (17)
C13—Sn1—Cl1	95.58 (5)	C15—C16—H16	119.8
O1—Sn1—Cl1	178.06 (3)	C17—C16—H16	119.8
Sn1—O1—H1o	111.1 (19)	C16—C17—C18	119.86 (18)
Sn1—O1—H2o	113 (2)	C16—C17—H17	120.1
H1o—O1—H2o	101 (3)	C18—C17—H17	120.1
C19—N1—C23	118.57 (15)	C17—C18—C13	120.82 (18)
C30—N2—C27	118.00 (16)	C17—C18—H18	119.6
C6—C1—C2	118.67 (16)	C13—C18—H18	119.6
C6—C1—Sn1	119.99 (12)	N1—C19—C20	123.95 (17)
C2—C1—Sn1	121.17 (13)	N1—C19—H19	118.0
C1—C2—C3	120.32 (18)	C20—C19—H19	118.0
C1—C2—H2	119.8	C21—C20—C19	118.14 (17)
C3—C2—H2	119.8	C21—C20—H20	120.9
C4—C3—C2	120.23 (19)	C19—C20—H20	120.9
C4—C3—H3	119.9	C20—C21—C22	119.97 (17)
C2—C3—H3	119.9	C20—C21—H21	120.0
C5—C4—C3	119.82 (17)	C22—C21—H21	120.0
C5—C4—H4	120.1	C23—C22—C21	118.24 (17)
C3—C4—H4	120.1	C23—C22—C24	120.29 (18)
C4—C5—C6	120.27 (18)	C21—C22—C24	121.47 (17)
C4—C5—H5	119.9	N1—C23—C22	121.12 (16)
C6—C5—H5	119.9	N1—C23—C27	119.92 (15)
C5—C6—C1	120.67 (17)	C22—C23—C27	118.95 (16)
C5—C6—H6	119.7	C25—C24—C22	120.82 (18)
C1—C6—H6	119.7	C25—C24—H24	119.6
C12—C7—C8	118.48 (16)	C22—C24—H24	119.6
C12—C7—Sn1	120.79 (13)	C24—C25—C26	121.20 (17)
C8—C7—Sn1	120.45 (13)	C24—C25—H25	119.4
C9—C8—C7	120.42 (19)	C26—C25—H25	119.4
C9—C8—H8	119.8	C28—C26—C27	117.91 (18)
C7—C8—H8	119.8	C28—C26—C25	122.01 (18)
C10—C9—C8	120.2 (2)	C27—C26—C25	120.06 (18)
C10—C9—H9	119.9	N2—C27—C26	121.80 (17)
C8—C9—H9	119.9	N2—C27—C23	119.53 (15)
C11—C10—C9	120.17 (18)	C26—C27—C23	118.66 (16)
C11—C10—H10	119.9	C29—C28—C26	119.80 (18)
C9—C10—H10	119.9	C29—C28—H28	120.1
C10—C11—C12	119.95 (18)	C26—C28—H28	120.1
C10—C11—H11	120.0	C28—C29—C30	118.33 (19)
C12—C11—H11	120.0	C28—C29—H29	120.8
C11—C12—C7	120.72 (18)	C30—C29—H29	120.8
C11—C12—H12	119.6	N2—C30—C29	124.1 (2)
C7—C12—H12	119.6	N2—C30—H30	117.9
C18—C13—C14	118.56 (16)	C29—C30—H30	117.9
C18—C13—Sn1	120.95 (13)		
			
C13—Sn1—C1—C6	12.62 (16)	C18—C13—C14—C15	−0.1 (2)
C7—Sn1—C1—C6	−151.49 (13)	Sn1—C13—C14—C15	−177.11 (13)
O1—Sn1—C1—C6	−68.37 (14)	C13—C14—C15—C16	−0.3 (3)
Cl1—Sn1—C1—C6	110.98 (13)	C14—C15—C16—C17	0.2 (3)
C13—Sn1—C1—C2	−172.10 (14)	C15—C16—C17—C18	0.4 (3)
C7—Sn1—C1—C2	23.79 (17)	C16—C17—C18—C13	−0.8 (3)
O1—Sn1—C1—C2	106.90 (15)	C14—C13—C18—C17	0.7 (3)
Cl1—Sn1—C1—C2	−73.75 (14)	Sn1—C13—C18—C17	177.66 (13)
C6—C1—C2—C3	1.4 (3)	C23—N1—C19—C20	−0.2 (3)
Sn1—C1—C2—C3	−173.93 (15)	N1—C19—C20—C21	0.6 (3)
C1—C2—C3—C4	0.0 (3)	C19—C20—C21—C22	−0.3 (3)
C2—C3—C4—C5	−1.3 (3)	C20—C21—C22—C23	−0.4 (3)
C3—C4—C5—C6	1.2 (3)	C20—C21—C22—C24	179.42 (18)
C4—C5—C6—C1	0.2 (3)	C19—N1—C23—C22	−0.6 (2)
C2—C1—C6—C5	−1.5 (3)	C19—N1—C23—C27	179.62 (15)
Sn1—C1—C6—C5	173.91 (13)	C21—C22—C23—N1	0.9 (3)
C1—Sn1—C7—C12	−78.22 (15)	C24—C22—C23—N1	−178.92 (16)
C13—Sn1—C7—C12	116.81 (13)	C21—C22—C23—C27	−179.32 (16)
O1—Sn1—C7—C12	−160.15 (13)	C24—C22—C23—C27	0.8 (3)
Cl1—Sn1—C7—C12	18.72 (13)	C23—C22—C24—C25	0.1 (3)
C1—Sn1—C7—C8	108.01 (14)	C21—C22—C24—C25	−179.75 (18)
C13—Sn1—C7—C8	−56.97 (16)	C22—C24—C25—C26	−0.7 (3)
O1—Sn1—C7—C8	26.08 (14)	C24—C25—C26—C28	−178.24 (19)
Cl1—Sn1—C7—C8	−155.05 (14)	C24—C25—C26—C27	0.4 (3)
C12—C7—C8—C9	−1.0 (3)	C30—N2—C27—C26	0.6 (3)
Sn1—C7—C8—C9	172.95 (15)	C30—N2—C27—C23	−178.48 (16)
C7—C8—C9—C10	0.2 (3)	C28—C26—C27—N2	0.1 (3)
C8—C9—C10—C11	1.2 (3)	C25—C26—C27—N2	−178.60 (17)
C9—C10—C11—C12	−2.0 (3)	C28—C26—C27—C23	179.23 (16)
C10—C11—C12—C7	1.2 (3)	C25—C26—C27—C23	0.5 (3)
C8—C7—C12—C11	0.2 (3)	N1—C23—C27—N2	−2.2 (2)
Sn1—C7—C12—C11	−173.66 (13)	C22—C23—C27—N2	178.03 (16)
C1—Sn1—C13—C18	66.35 (15)	N1—C23—C27—C26	178.62 (16)
C7—Sn1—C13—C18	−127.99 (13)	C22—C23—C27—C26	−1.1 (2)
O1—Sn1—C13—C18	147.42 (13)	C27—C26—C28—C29	−0.6 (3)
Cl1—Sn1—C13—C18	−30.67 (13)	C25—C26—C28—C29	178.06 (19)
C1—Sn1—C13—C14	−116.71 (13)	C26—C28—C29—C30	0.4 (3)
C7—Sn1—C13—C14	48.95 (15)	C27—N2—C30—C29	−0.9 (3)
O1—Sn1—C13—C14	−35.64 (13)	C28—C29—C30—N2	0.4 (3)
Cl1—Sn1—C13—C14	146.27 (12)		

### Hydrogen-bond geometry (Å, °)

**Table:**

*D*—H···*A*	*D*—H	H···*A*	*D*···*A*	*D*—H···*A*
O1—H1*o*···N1	0.84 (1)	1.91 (1)	2.716 (2)	159 (3)
O1—H2*o*···N2	0.84 (1)	2.03 (2)	2.757 (2)	144 (3)
